# Urinary Tract Infection Caused by the Novel Pathogen, Lactococcus Garvieae: A Case Report

**DOI:** 10.7759/cureus.9462

**Published:** 2020-07-29

**Authors:** Ezza Fatima Tariq, Yusra Irshad, Heyyan B Khalil, Aemen S Khakwani, Usman A Khan

**Affiliations:** 1 Internal Medicine, Nishtar Medical University/Hospital, Multan, PAK; 2 Nephrology, Oklahoma University Health Sciences Center, Oklahoma City, USA; 3 Internal Medicine, Kulsoom International Hospital, Islamabad, PAK; 4 Internal Medicine, University of Oklahoma Health Sciences Center, Oklahoma City, USA; 5 Internal Medicine, Suburban Community Hospital, East Norriton, USA; 6 Internal Medicine and Nephrology, University of Oklahoma Health Sciences Center, Oklahoma City, USA

**Keywords:** aquaculture, amoxicillin – clavulanate, urinary tract infection (uti), lactococcus garvieae (l. garvieae)

## Abstract

*Lactococcus garvieae* is a part of the genus *Lactococcus* which was previously a part of the genus *Streptococcus*. It has been associated with serious diseases in aquaculture. However, human infections are rare. The most common presentation in humans is infective endocarditis. Urinary tract infection (UTI) is a unique presentation of this pathogen.

We report the case of a 70-year-old male with a past history of benign prostatic hyperplasia who presented with confusion. Urinalysis confirmed the growth of *L. garvieae*. A seven-day treatment course of amoxicillin-clavulanate successfully treated the patient.

The risk factors for acquiring the *L. garvieae* infections include contact with seafood or aquaculture. Other risk factors include immunosuppression, gastric acid suppression, and anatomical or physiological defect in the gastrointestinal tract. Special tests, such as VITEK^®^ 2, API® 32 strep system, 16S rRNA, or polymerase chain reaction (PCR) testing, are needed for its diagnosis. Hence, we suggest underreporting of the infection may be possible.

It is a novel cause of UTI, we suggest a high index of suspicion should be kept, especially in people with associated risk factors or exposure to seafood.

## Introduction

*Lactococcus* genus, originally part of the Streptococcus genus, was made an independent genus in 1985. This group has eight species and subspecies [1]. Included in this genus, *Lactococcus garvieae *(*L. garvieae*) and *Lactococcus lactis* (*L. lactis*) are associated with human infection *L. garvieae* causes hemorrhagic sepsis in the aquaculture and is associated in the pathogenesis of bovine mastitis and pneumonia in pigs. *L. garvieae* can be found in a vast variety of environments due to its ability to adapt easily. It has been isolated from aquaculture, including sewage waters and rivers. It is also found as a contaminant of food material. It has been isolated from raw milk, cheese, vegetables, cereals, and meat [2].

The first case of human infection was observed in 1991 [3-4]. Elliot et al. reported human infections caused by *L. garvieae* and, to a lesser extent, by *L. lactis* [4]. Human infections by *L. garvieae* are rare [5]. A total of 19 cases of human infection by *L. garvieae* were reported until 2015 [6]. However, infections in humans are associated with high morbidity and around 25% mortality rate. In humans, cases of *L. garvieae* causing infective endocarditis, bacteremia, secondary peritonitis, liver abscess, diverticulitis, and spondylodiscitis have been observed. *L. garvieae* associated urinary tract infection is relatively a rare presentation. Previously, three cases have been reported [1, 6-7]. We report a novel case of urinary tract infection caused by *L. garvieae* as an isolated pathogen.

## Case presentation

A 70-year-old male white male with a past medical history of esophageal cancer, status-post chemotherapy and radiation therapy, hypertension, and benign prostatic hyperplasia presented to the hospital with a history of confusion and subjective feeling of warmth. The patient was accompanied by his wife who reported the patient had been confused for the past two days. He was febrile with a temperature of 100.9°F. Otherwise, he was hemodynamically stable with normal vitals on the presentation. Initial laboratory studies showed mild leukocytosis of 14,000 (normal: 4,500 to 11,000/L) with a left shift primarily neutrophilic. Urinalysis revealed 180 white blood cells/high power field (WBC/HPF) (normal: 2 - 5 WBC/HPF) and 2+ leukocyte esterase (normal: 0). The complete blood count (CBC) was 5 x 10^12^/L (normal: 4.7 to 6.1 x 10^12^/L. The hemoglobin level was 13 mg/dL (normal: 13.5 - 17.5 mg/dL). The platelet count was 172 x 10^9^/L. The blood urea nitrogen (BUN) was 28 mg/dL (normal: 8 - 20 mg/dL). The creatinine level was 1.8 mg/dL (normal: 0.84 to 1.21 mg/dL). The C-reactive protein (CRP) level was 89 mg/dL (normal: below 3.0 mg/L). The erythrocyte sedimentation rate (ESR) was 60 mm/hr (normal: 0 - 22 mm/hr). The serum electrolyte level revealed a sodium level of 138 mEq/L (normal: 135 - 145 mEq/L), a potassium level of 4 mEq/L (normal: 3.7 - 5.2 mEq/L), and a chlorine level of 89 mEq/L (normal: 96 - 106 mEq/L). Two sets of blood cultures were sent which subsequently were negative. The urine cultures grew more than 100,000 colony-forming units per milliliter of *L. garvieae*. The sample was sent to the National Microbiologist Reference Laboratory for identification of the pathogen by polymerase chain reaction (PCR), as we were unable to do it in our hospital laboratory. On Day 3 of admission, there was a considerable improvement in the patient's mentation. The patient was empirically started on ceftriaxone, 1 gm intravenous daily, which was later changed to Augmentin, shifting from intravenous to the oral dose. Upon further interviewing, the patient reported no exposure to raw fish or unpasteurized dairy products; however, long-term ranitidine use was reported. He was treated with amoxicillin-clavulanate, 1 gram twice per day for a total of seven days, and then discharged home. The patient was readmitted to the hospital three weeks later after a fall and syncopal episode secondary to orthostasis and dehydration. Repeat urine analysis was negative for any leukocytes or nitrites. Repeat cultures during the second admission were negative for any bacterial growth. 

## Discussion

*L. garvieae* is a gram-positive, catalase-negative, facultatively anaerobic cocci that can exist in a singular form, pairs, or a chain. It uses carbohydrates to produce lactic acid [1].

Infections in humans is often associated with the handling of fish, as well as unpasteurized milk [1, 8]. There are certain risk factors that predispose a patient to acquire infection by *L. garvieae*. Certain risk factors, including immune deficiencies, diverticulosis, and gastric ulcers, predispose a patient to infection by *L. garvieae*. Chronic acid suppression by the use of a proton pump inhibitor (PPI) or H_2_ receptor blocker has also been suggested as a risk factor [6].

Urinary tract infection is relatively a rare presentation of an *L. garvieae* infection. According to our literature search, this is the fourth case being reported. The previous cases presented as spontaneous infection, post-transurethral resection of the prostate (TURP), or as catheter-induced infection [1, 6-7]. Two of these patients reported a history of using PPIs or H_2 _receptor blockers [1, 6]. Similarly, our patient reported long-term use of ranitidine. This association of this risk factor with *L. garvieae* infection is reinforced by our case.

Methods to identify *L. garvieae* include special tests like VITEK®2, API® 32 strep system, 16S rRNA, or PCR testing [9]. These tests can be used to successfully differentiate *L. garvieae* from enterococci. These tests are not available in many centers worldwide. Therefore, it is highly likely that the current incidence of *L. garvieae* infections is an underestimation. Clindamycin resistance of *L. garvieae* is further helpful to differentiate it from *L. lactis* [1, 9].

The antibiotics used against *L. garvieae* include amoxicillin, ceftriaxone, and vancomycin used as monotherapy or in combination with gentamicin [9]. Our patient improved after seven days of treatment with amoxicillin-clavulanate. Repeat urine culture after three weeks was found to be negative, confirming a successful treatment.

*L. garvieae* is an infrequent cause of urinary tract infection in humans. Its similarity with other species, like *L. lactis* and *Enterococcus*, makes the diagnosis a difficult task. Moreover, in many cases, it is found to cause co-infections with other pathogens. The diagnosis requires specialized and expensive equipment which is not readily available in most parts of the world. Hence, we suggest there might be under-reporting of cases. The way *L. garvieae* cause pathogenesis is poorly known as well.

This underscores the need for further research on this pathogen and diseases caused by it. Moreover, an index of suspicion should be kept in cases with a history of seafood exposure or any of the aforementioned risk factors in a patient with a urinary tract infection.

## Conclusions

Common micro-organisms associated with urinary tract infections include *E. coli*, *Klebsiella*, and *Proteus* species. Physicians in clinical practice are more likely to treat empirically for these pathogens. However, in the case of treatment resistance and culture-negativity for these pathogens, other causative agents must be kept on the differential diagnosis list. The patient should be enquired about the mentioned risk factors and sample tested for *L. garvieae* growth as well.

We report this unique case where the patient acquired an *L. garvieae* urinary tract infection, possibly due to the risk factor of long-term acid suppression medication. It is also an opportunistic pathogen and can infect immune-compromised patients. As seen in the case we present, the patient had a history of esophageal cancer treated with chemotherapy and radiotherapy. We want to emphasize and bring attention to physicians and infectious disease specialists the potential of *L. garvieae* as an emerging cause of UTI.
